# Using predictive process monitoring to assist thrombolytic therapy decision-making for ischemic stroke patients

**DOI:** 10.1186/s12911-020-1111-6

**Published:** 2020-07-09

**Authors:** Haifeng Xu, Jianfei Pang, Xi Yang, Mei Li, Dongsheng Zhao

**Affiliations:** 1 Information Center, Academy of Military Medical Sciences, Beijing, People’s Republic of China; 2 Medical Service Department, General Hospital of Xinjiang Military Region, Urumchi, People’s Republic of China; 3 China Stroke Data Center, Beijing, People’s Republic of China

**Keywords:** Predictive process monitoring, Clinical decision support, Clinical guideline, Stroke thrombolytic therapy

## Abstract

**Background:**

Although clinical guidelines provide the best practice for medical activities, there are some limitations in using clinical guidelines to assistant decision-making in practical application, such as long update cycle and low compliance of doctors with the guidelines. Driven by data of actual cases, process mining technology provides the possibility to remedy these shortcomings of clinical guidelines.

**Methods:**

We propose a clinical decision support method using predictive process monitoring, which could be complementary with clinical guidelines, to assist medical staff with thrombolytic therapy decision-making for stroke patients. Firstly, we construct a labeled data set of 1191 cases to show whether each case actually need thrombolytic therapy, and whether it conform to the clinical guidelines. After prefix extraction and filtering the control flow of completed cases, the sequences with data flow are encoded, and corresponding prediction models are trained.

**Results:**

Compared with the labeled results, the average accuracy of our prediction models for intravenous thrombolysis and arterial thrombolysis on the test set are 0.96 and 0.91, and AUC are 0.93 and 0.85 respectively. Compared with the recommendation of clinical guidelines, the accuracy, recall and AUC of our predictive models are higher.

**Conclusions:**

The performance and feasibility of this method are verified by taking thrombolytic decision-making of patients with ischemic stroke as an example. When the clinical guidelines are not applicable, doctors could be provided with assistant decision-making by referring to similar historical cases using predictive process monitoring.

## Background

In clinical practice, doctors often need to make decisions based on their experience of diagnosis and treatment, as well as the specific situation of each patient. For example, they want to know whether thrombolysis therapy is necessary for patients with ischemic acute stroke. According to reference [1], the general doctor’s judgment of thrombolysis for stroke patients is not accurate, and the misperception for the rate of fatal intracranial hemorrhage using rt-PA may interfere with their willingness to endorse this treatment. Clinical guidelines (CGs) offer the best practice in medical activities and play an important role for improving medical quality as well as reducing risks. However, evidence in CG is essentially a form of statistical knowledge, which is used to capture the generalities of patient groups, rather than the peculiarities of a specific patient. Thus, several conditions are usually implicitly assumed by experts building a CG [2]:

(i) ideal patients, i.e., patients that have ‘just the single’ disease considered in the CG (thus excluding the concurrent application of more than one CG), not presenting rare peculiarities or side-effects;
(ii) ideal physicians executing the CG, i.e., physicians whose basic medical knowledge always allow them to properly apply the CGs to specific patients;
(iii) ideal context of execution, so that all of necessary resources are available.

However, influenced by various factors (such as economic ability, cultural concepts, etc.), doctors usually have low compliance with clinical guidelines in real medical environment [3, 4]. These factors need to be fully considered for providing clinical decision support.

Due to the application of medical information system such as EMR, a large number of valuable historical diagnostic and therapeutic data have been stored. Based on mining and analyzing these medical data of related patients in the past, it has practical application value for medical decision support. Many studies analyze big data to make prediction, which can be divided into two categories [5]: one is supervised method for specific application purposes to train and generate predictive models, such as classification, regression, deep neural network, etc.; the other is unsupervised method by measuring the distance between patients, establishing similar groups of patients, and predicting the health status of target patients with the characteristics of similar groups. Although both of them need to pre-process data such as variable selection and dimensionality reduction, the former often trains a prediction model with higher accuracy, and the latter has a broader prediction ability [6].

As an active branch of medical knowledge engineering and artificial intelligence research, clinical decision support system (CDSS) is always the focus of study and application. However, what can be accepted by doctors and put into clinical use is few in the current CDSS. The main reason is that current CDSS relies too much on clinical medicine knowledge inference rules (CG are essentially medical rules), while some new rules are difficult to be obtained and represented [7]. Due to paying little attention to diversity, variability and uncertainty factors of disease, these systems cannot help doctors in the case of complex patient and disease. Some problems of the rule-based reasoning (RBR) system can be solved to some extent by the method of case-based reasoning (CBR). CBR solves problems by searching and matching with features of previous cases. But there are some shortcomings when CBR is used only, for example, it is difficult to express the deep domain knowledge; case retrieval and similarity matching algorithm should be further improved. Through combining with new technologies and methods of academic subjects, CBR and RBR can play their respective advantages by integrating, improving the application effect of CDSS. Rossille et al. [8] present the overall framework of CDSS based on CBR and RBR, while they do not define the specific similarity metric.

Clinical processes can be seen as complex symbolic sequences, i.e., a sequence of events each carrying a data payload consisting of event attributes [9]. Both the sequence of medical events (control flow) and the attributes value of events (data flow) have a significant impact on medical outcomes. For example, a doctor may perform a certain type of surgery only if it is preceded by a preoperational screening; the event attributes include the age of the patient or the amount of glucose in a blood sample. Traditionally, the studies of medical data mining focus on cross-sectional data such as diagnosis, symptoms, examination, past history, drugs, surgery and so on, while the time information is seldom involved [6]. Process mining technology involves time factor on the basis of data mining, and is driven by the data of actual cases. It records specific activity information through event logs to reflect the actual business execution history and predict the status of next activities [10].

Taking thrombolytic decision-making of stroke patients for example, this paper proposes a method using predictive process monitoring, which could be complementary with clinical guidelines. When the clinical guidelines are not applicable, it could provide decision support for doctors by referring to related historical cases, and the accuracy and feasibility of this method are verified. The rest of this paper is structured as follows. Section 2 shows background knowledge including predictive process monitoring and clinical guidelines for acute ischemic stroke. The proposed framework is described in Section 3. Section 4 illustrates the data set, experimental design and results. Section 5 discusses related work and Section 6 draws conclusions.

### Preliminary Knowledge

#### Clinical guidelines for acute ischemic stroke

Cerebral Vascular Disease (CVD) is the leading cause of disability and death in China [11]. It can be classified into two categories: ischemic stroke and hemorrhagic stroke, which accounts for about 70% and 25% respectively. Studies of Evidence-based medicine (EBM) have proven that the efficient diagnosis and treatment for acute stroke played an important role in reducing disability, mortality and recurrence rates [12]. Based on clinical evidence and consensus of experts, clinical guidelines provide the best practice for each treatment method, with different strength of recommendation and quality of evidence. American Stroke Association has started the Get With The Guidelines (GWTG), which is a quality improvement program designed to close the treatment gap in stroke, by promoting consistent adherence to the latest scientific treatment guidelines, and it has succeed in achieving measurable improvements of outcome [13].

The most effective treatment for acute ischemic stroke (AIS) is revascularization within a time window, including intravenous thrombolysis and intravascular therapy. The commonly used thrombolytic drugs in China are recombinant tissue plasminogen activator (rt-PA) and urokinase. At present, several clinical trials suggest that the time window for effective treatment is within 4.5 h or 6 h. The indications, contraindications and relative contraindications of different thrombolytic drugs and time windows are clearly defined in the clinical guidelines. For example, indications and contraindications for rt-PA intravenous thrombolysis within 3 h [14] are shown in the Table 1.

**Table 1:** Indications and Contraindications for rt-PA intravenous thrombolysis within 3 h

Type	No.	Eligibility Recommendations
Indications	1	Symptoms of neurological impairment caused by ischemic stroke
	2	Within 3 h of ischemic stroke symptom from onset or patient last known well or at baseline state.
	3	Patients older than 18 years
	4	Informed consent signed by patient or family member
Contraindications	1	Intracranial hemorrhage
	2	With history of intracranial hemorrhage
	3	Stroke or severe head injury within 3 months
	4	Intracranial neoplasms or giant intracranial aneurysms
	5	Intracranial or intraspinal surgery within 3 months
	6	Received large surgery within 2 weeks
	7	Gastrointestinal bleed or urinary system hemorrhage within 3 weeks
	8	Associated with active visceral hemorrhage
	9	Associated with aortic arch dissection
	10	Arterial punctures with difficult to hemostasis in the past week
	11	Blood pressure > 180/100 mmHg
	12	Platelet count < 100* 10^9^/L
	13	Treatment with low-molecular-weight heparin within 24 h
	14	international normalized ratio (INR) > 1.7 or Prothrombin time (PT) > 15 s
	15	Treatment with thrombin inhibitors or factor Xa inhibitors within 48 h, or abnormal laboratory examinations
	16	Blood glucose < 2.8 mmol/L or blood glucose > 22.22 mmol/L
	17	Large area of head infarction

These indications and contraindications provide a basis for clinicians to screen thrombolytic patients, and there are some decision support systems based on clinical guidelines. However, due to the complexity of medicine, clinical guidelines just offer general treatment recommendations for people with disease in statistical perspective. Furthermore, clinical guidelines often need several years to be built or updated, even there are contradictions between different guidelines. At the same time, for some patients with complex complications, clinical guidelines are presented such as ‘evaluating risk and benefit’ or ‘further clinical research is required’, so doctors have to judge by their own experience. Therefore, these factors could lead to low compliance rate of doctors with clinical guidelines [3, 4], which may interfere the popularization of appropriate techniques for stroke treatment and increase unnecessary mortalities and disabilities.

#### Predictive process monitoring

Clinical process consists of a set of activities, including prevention, diagnostic, therapy and rehabilitation, to improve the health status of patients. The effectiveness of these processes often determines the quality of medical services. Besides, with the development of medical technology, the complexity of medical process is increasing. There is always the need to reduce the cost of health care, decrease patient’s waiting times, improve resources productivity, and increase processes transparency [10].

Due to the application of medical information system, event logs are generated at every step of the medical treatment [15]. Process mining techniques use event data to discovery process models, to check the conformance with predefined process models, and to improve such models with information about bottlenecks, decisions, and resource usage [16].

The starting point of process monitoring are event records representing the execution of activities in a business process. An event record has a number of attributes. For example, the event class (activity name) specifying which activity the event refers to, the timestamp specifying when did the event occur, and the case id indicating which case of the process generated this event. An event record may carry additional attributes in its payload, including event attributes and case attributes. Each event or case attribute can be of numeric, categorical, or of textual data type. In this paper, we use natural language processing technology to convert textual data type into numerical or categorized variables for predicting. Possible event and case attributes as well as their types are presented in Table 2.

**Table 2:** Data attributes in the event log

	Type	Example
Case (static)	Categorical	Patient’s gender
	Numeric	Patient’s age
	Textual	Description of the application
Event (dynamic)	Categorical	Activity, resource
	Numeric	Amount paid
	Textual	Patient’s medical history

**Fig. 1:**
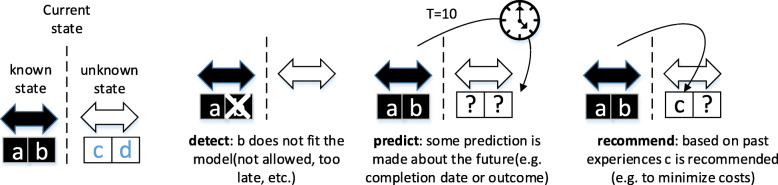
Three operational support activities: detect, predict, and recommend

Most process mining techniques work on ‘post mortem’ event data, i.e., they analyze events that belong to cases which have already completed. Today, however, many data sources are updated in (near) real-time and sufficient computing power is available to analyze data when they come into being. Therefore, process mining should not be restricted to off-line analysis and can also be used for online operational support. Figure 1 shows three process mining activities related to operational support: detect, predict, and recommend. Consider a case for which activities a and b have been executed, in the state after observing partial trace *δ* = < a, b > describing the known past of the case, the future of the case is not known yet.

(i) Detect. This activity compares the partial trace *δ* with some normative models, e.g., a process model or an LTL constraint. If b was not allowed after a, an alert would be generated.

(ii) Predict. This activity makes statements about the events following *δ*. For example, the expected completion time could be predicted by comparing the current case to similar cases that were handled in the past.

(iii) Recommend. Recommendations guide the user in selecting the next activity after *δ*. For example, based on historic information, it could recommend to execute activity c next to minimize costs or flow time.

Traditional process monitoring techniques provide dashboards or reports showing the recent performance of a business process in terms of key performance indicators such as mean execution time, resource utilization, error rate and so on. However, predictive (business) process monitoring refers to the act of making predictions about the future state of ongoing cases of a business process, based on their incomplete execution traces and logs of historical (completed) traces [17].

Motivated by the increasingly pervasive availability of fine-grained event data about business process executions, the problem of predictive process monitoring has received substantial attention in recent years. For example, Aalst et al. in [18] proposed a framework for operational support using process mining and details a coherent set of approaches that focuses on time information. The authors in [19] presented an approach to analyze event logs in order to predictively monitor business goals defined in the form of linear temporal logic rules, during business process execution. In [20], the authors designed a predictive process monitoring framework, taking into account both the sequence of events and data attributes associated to these events, for estimating the probability that a given predicate will be fulfilled upon completion of a running case.

With respect to the broader literature on machine learning, predictive process monitoring corresponds to a problem of early sequence classification [9]. In other words, given a set of labeled sequences, the goal is to build a model that for a sequence prefix predicts the label when this prefix completed. While there are substantial literature on the problem of sequence classification for simple symbolic sequences (e.g., sequences of events without payloads), there is a lack of proposal addressing the problem for complex symbolic sequences (i.e., sequences of events with payloads). The problem of outcome-oriented predictive process monitoring can be seen as an early classification over complex sequences where each element has a timestamp, a discrete attribute referring to an activity, and a payload made of a heterogeneous set of other attributes.

## Methods

**Fig. 2:**
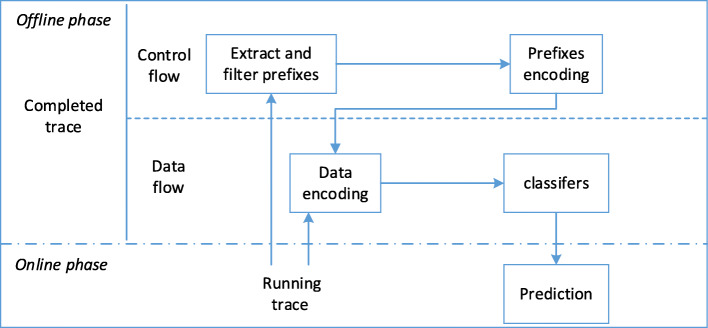
Framework of predictive process monitoring

The framework proposed consists of two phases: offline, to train a prediction model based on historical cases, and online, to make predictions on running process cases. The function of each phase is shown in Fig. 2. There are four steps in the offline phase. Firstly, given an event log, case prefixes are extracted and filtered. Next, the selected prefixes in control flow and attributes in data flow are encoded for classification. Finally, some supervised learning algorithms are used to train classify models. The online phase concerns the actual prediction for a running trace, by reusing the classifiers built in the offline phase.

The original data features of patients include age, sex, pulse, blood pressure, blood glucose, hypertension, symptom, etc. These data features are involved in structured data and unstructured data, which are stored in HIS, LIS, PACS, EHR or other information systems. Data extraction and conversion are needed at first. We use Beautiful Soup [21], which is a Python library for natural language processing, to extract information from unstructured text. For example, the time from onset to consultation is abstracted from patient’s complaint; neurological impairment score tested in FAST-ED [22] is computed based on history of present illness; history of stroke and intracranial hemorrhage are extracted from history of past illness.

### Prefix extraction and filtering

**Fig. 3:**
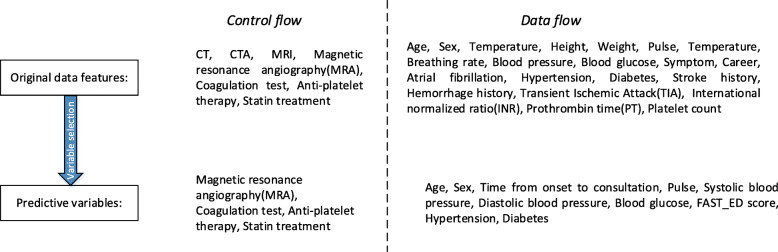
Original data features and predictive variables

Using a prefix log ensures that our training data is comparable to the testing data. For example, in a complete trace consisting of a total of 5 events, we could consider up to 4 prefixes: the partial trace after executing the first event, the partial trace after executing the first and the second event, and so on. Since the large number of prefixes as compared to the number of traces slows down and causes bias in the training of the prediction models, it is common to consider prefixes up to a certain number of events only. For example, Leontjeva et al. [9] and Di Francescomarino et al. [20] limit the maximum prefix length to 20 and 21 respectively. For our data set, there are 7 events in control flow, which are list in Fig. 3. After prefix filtering according the importance of data features, we select 4 events in the acute period (within 24 h from onset) as prefix log, i.e., magnetic resonance angiography (MRA), coagulation test, anti-platelet therapy, statin treatment.

### Sequence encoding

In order to train a classier, all prefix traces need to be represented as fixed length feature vectors. With event executing, additional information about the case becomes available, while each trace should still be represented in the same number of features. To solve this problem, a sequence encoding method can be thought of as a combination of a trace abstraction technique and a set of feature extraction functions for each data attribute. In this paper, we encode a trace (prefix) according to the frequency of the occurrence of sequence patterns. Case attributes are represented by static coding; event attributes are represented by last state coding [19, 20], i.e., only the last available snapshot of the data is used. As the size of the feature vector does not depend on the length of the trace, the last state encoding can be used with traces of different lengths.

For example, given the trace t1 = <A, B, C, D, B > and trace t2 = <A, C, D, E>, we can represent the alphabet of the events as an ordered vector L = <A, B, C, D, E>. In this case, the control flow of t1 will be encoded as a vector of frequencies < 1,2,1,1,0>, and t2 will be < 1,0,1,1,1>. If the payload of event B (blood glucose) are 5.1 and 8.3 respectively, the data attribute of B is 8.3.

### Classification algorithm

The existing predictive process monitoring methods are experimented with different classification algorithms. The most popular choice is decision tree (DT), which has obvious benefits in terms of the interpretability of the results. Another popular method is random forest (RF), which usually achieves better prediction accuracy than a single decision tree, and Cui et al. [23] has proved the interpretability of the results of RF. Additionally, Leontjeva et al. [9] experimented with support vector machines (SVM) and generalized boosted regression models (GBM), but found that their performance is inferior to RF. Therefore, we choose DT and RF implemented in sklearn [24] library as the classification algorithm. Minimum sample leaf is chosen in DT and RF as hyper-parameter, and class weight is set to balanced. To train a model in RF, 10 basic classifiers are used.

## Experiment

### Data set

From the Electronic Medical Record (EMR) system of a large general hospital in China, we extracted discharged patients who were admitted to the Department of Neurology between January 2013 and July 2019. These patients are diagnosed with acute ischemic cerebral infarction, and within 24 h from onset to consultation. Patients who voluntarily request to be discharged from hospital or wake-up stroke patients are excluded. SQL statements and Python scripts are used to retrieve cases from database and medical documents respectively, and 1191 qualified cases are selected. It should be noted that the correctness of treatment for each case has been discussed and confirmed by medical experts. The composition of each type of treatment in the data set is shown in Table 3.

**Table 3:** Therapeutic method in the data set

Intravenous thrombolysis		Endovascular treatment			Others
rt-PA	urokinase	Mechanical Thrombectomy	Arterial thrombolysis		
			rt-PA	urokinase	
123	5	15	86	59	903

According to the data items involved in the clinical guidelines [14, 25], only relatively important attributes indicating by variance selection method are used as predictive variables in our models, i.e., the variance of each feature is calculated first, and then the features whose value is greater than the threshold are selected. The original data features and predictive variables selected are list in Fig. 3.

Part of the experimental dataset before normalization is shown in Table 4. OTC is the time from onset to consultation recorded in the patient’s complaint; FAST is the score of FAST_ED test; MRA means the event of magnetic resonance angiography; coag is the coagulation test; statin means the statin treatment.

**Table 4:** A fragment of data set

ID	Practical thrombolysis	Comply with the guidelines	Age	OTC	Glucose	Platelet count	Blood pressure	FAST	MRA	Coag	Statin	…
1	1	0	75	3	7.9	550	150/80	2	0	1	1	
2	0	1	88	5	11.52	200	120/70	1	1	1	0	
3	0	0	80	12	16.69	110	140/90	1	0	1	0	
4	1	1	56	8	6.48	430	130/76	2	0	0	1	
5	0	1	70	7	5.34	230	200/90	1	1	1	0	
…												

### Experimental design

**Fig. 4:**
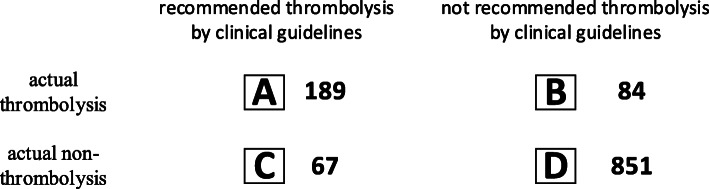
Checking results of the data set

After choosing the predictive variables shown in Fig. 3, it is necessary to label each instance whether each patient followed to the clinical guidelines and whether each patient received thrombolytic therapy. Follow guidelines(A and D) means that if clinical guidelines recommend thrombolysis then patients actually get thrombolytic therapy(A), and vice versa(D); otherwise, it does not conform to clinical guidelines(B and C). Clinical guidelines recommend thrombolysis for patients who satisfy the indications without contraindications. Based on the indications and contraindications of the clinical guidelines, we developed a rule checking program using Python to determine whether each case conformed to the recommendations of the guidelines, and the checking result has been confirmed by medical experts. The overall checkup results are shown in the Fig. 4.

There are 151 cases(B and C) in the experimental dataset that does not conform to the clinical guidelines, which account for 12.6% of the total cases. It indicates that the actual medical decision-making is not completely consistent with the recommendation from the clinical guidelines. In the experimental data set, there are 273 actual thrombolysis(A and B) and 918 Non-thrombolysis(C and D) cases. After proportional random sampling, we use the first part (80% of the traces) as training set, i.e., these traces are used as historical data to derive predictions. The remaining 20% dataset are used as the uncompleted traces (test set).

To evaluate the effectiveness of our approach, we classified prediction results in four categories, i.e., i) true-positive (T_P_: positive outcomes correctly predicted); ii) false-positive (F_P_: negative outcomes predicted as positive); iii) true-negative (T_N_: negative outcomes correctly predicted); iv) false-negative (F_N_: positive outcomes predicted as negative). The gold standard used as reference is the set of all instances with actual thrombolysis, which have been confirmed by medical experts. In our experiments, we can easily identify these instances. Accuracy in our context indicates how many times a prediction was correct:

$$ ACC=\frac{Tp+ Tn}{Tp+ Fp+ Tn+ Fn} $$

The recall defines how many positive outcomes are correctly predicted among all positive examples available:

$$ Recall=\frac{Tp}{Tp+ Fn} $$

On the other hand, the precision indicates how many positive outcomes are correctly predicted among all the outcomes predicted as positive:

$$ Precision=\frac{Tp}{Tp+ Fp} $$

F1 is defined in terms of harmonic mean of precision and recall:

$$ F1=\frac{2\ast Precision\ast Recal\mathrm{l}}{Precison+\mathrm{R} ecall} $$

AUC is the area under ROC (Receiver Operating Characteristic) curve. The horizontal axis of ROC is false positive rate (FPR) and the vertical axis is true positive rate (TPR). The larger of the AUC value, means the performance of classification is better.

## Results

The results drawn from the prediction models are compared with the actual thrombolysis cases(A and B) to evaluate the accuracy of the models. Because thrombolytic therapy for patients with ischemic stroke includes intravenous thrombolysis and arterial thrombolysis, two prediction (classification) models are generated. Secondly, thrombolysis and Non-thrombolysis are two separate decisions, so we should test their accuracy respectively. Thirdly, in order to evaluate the prediction effect of control flow, we compared the prediction models with control flow and without control flow. Moreover, we exploited DT and RF classification algorithms so as to improve the prediction performance. Lastly, clinical guidelines could be considered as another prediction model, so their ACC, precision, recall, and AUC can be calculated. The results on the test set by our predictive models are compared with those recommended by clinical guidelines(A and C), in order to assess whether the models are superior to those clinical guidelines.

We use a five-fold cross-validation method to evaluate the prediction performance. Compare with the actual results, the average accuracy of our prediction model (RF) on the test set is 0.96 and 0.91, the AUC is 0.93 and 0.85. Compared with the recommended results by clinical guidelines, the accuracy, recall and AUC of our predictive model are higher, which means our models are better at fitting the actual situation than the clinical guidelines. The precision for intravenous thrombolytic are also higher than recommendation from clinical guidelines, but the precision for arterial thrombolysis are lower than recommended by clinical guidelines. All of the experimental results are showed in Table 5**.**

**Table 5:** Experimental results on test set

Clinical application	Prediction model	ACC	Precision		Recall		F1		AUC
			throm	Non-throm	throm	Non-throm	throm	Non-throm	
Intravenous thrombolysis	DT without control flow	0.87	0.49	0.96	0.77	0.89	0.60	0.92	0.83
	DT with control flow	0.93	0.64	0.99	0.96	0.92	0.77	0.96	0.94
	RF without control flow	0.88	0.51	0.99	0.93	0.87	0.65	0.93	0.90
	RF with control flow	0.96	0.82	0.98	0.88	0.97	0.85	0.98	0.93
	Clinical guidelines	0.91	0.64	0.95	0.68	0.95	0.66	0.95	0.81
Arterial thrombolysis	DT without control flow	0.74	0.29	0.93	0.62	0.76	0.39	0.83	0.69
	DT with control flow	0.85	0.48	0.97	0.82	0.86	0.60	0.91	0.84
	RF without control flow	0.82	0.39	0.92	0.54	0.86	0.45	0.89	0.70
	RF with control flow	0.91	0.65	0.96	0.76	0.93	0.70	0.95	0.85
	Clinical guidelines	0.89	0.73	0.91	0.36	0.98	0.48	0.94	0.67

Since we train prediction models based on actual historical data, the higher recall could indicates doctors are inclined the benefit got from thrombolysis therapy, while the clinical guidelines perhaps are concerned with the risk of bleeding. In practical application, our models could remind medical staff occasions where thrombolysis therapy is practically possible but does not recommended by the clinical guidelines. For example, there are 75 cases recommended by our models but not by clinical guidelines, and 30 of them are with contraindications. The performance for intravenous thrombolysis are better than for arterial thrombolysis, which may be due to the time window is longer(0–24 h), and doctors might have to consider more factors in making decisions.

The performance of DT with control flow is better than DT without control flow, could be because we added 4 events: anti-platelet therapy, statin treatment, MRA, and coagulation test. Their proportions in the thrombolysis group and the non-thrombolysis group are significantly different (CI 95%, *P* < 0.01). The distribution of thrombolysis and non-thrombolysis for each medical activity are shown in the Table 6.

**Table 6:** Proportions of each medical activity

	Anti-platelet		Statin		MRA		Coagulation	
	throm	Non-throm	throm	Non-throm	throm	Non-Throm	throm	Non-throm
Intravenous thrombolysis	8/128	396/918	10/128	177/918	7/128	204/918	44/128	746/918
Arterial thrombolysis	17/145	712/918	16/145	500/918	19/145	287/918	78/145	849/918

## Discussion

At present, the building and update of clinical guidelines usually takes a long time. However, CG cannot cover all of the medical problems, even there are some contradictions between different clinical guidelines. In order for clinical practice guidelines to be effective, they need to be integrated with the care flow and provide patient-specific advice when and where needed. Hence, their formalization as computer-interpretable guidelines (CIGs) makes it possible to develop CIG-based decision-support systems (DSSs), which have a better chance of impacting clinician behavior than narrative guidelines. However, how does the patients’ personal context affect decision making, as well as developing process learning methods that could mine relationships between process context, need to be investigated further [26].

The past decade has seen an explosion in the amount of digital information stored in EMR. Over the same period, the machine learning community has seen widespread advances in the field of deep learning. A variety of deep learning techniques and frameworks are being applied to several types of clinical applications including information extraction and outcome prediction. For example, Long Short-Term Memory (LSTM) could accept sequence as input and produce better performance than DT or RF. However, a much larger data set is required for LSTM. On the other side, although in many circumstances predictive models are improved by using deep learning methodologies, model transparency is of utmost importance to clinical applications. Practitioners often do not take advice from clinical decision support tools that they do not understand [5].

The patient similarity analysis provides a general-purpose computer assistant clinical decision support framework, using the patient distance assessment. Up to date, this method has been initially approved in many medical domains such as cancer, endocrine diseases and heart diseases. Unlike the supervised methods used in building prediction models, patient similarity analysis adopts unsupervised or semi-supervised methods, then calculates the similarity between concepts according to the meaning of clinical concepts, to obtain the distance between patients, and finally predicts the runtime case using the most similar cluster of patients. At present, most of the studies in this field only use the data in static perspective for similarity analysis, lacking of time series characteristics, which cannot fully reflect the dynamic similarity between patients [27].

## Conclusions

Although the overall performance of our models is better than that of clinical guidelines, it is not to replace clinical guidelines, but to provide a new reference tool for doctors. The method we presented synthesizes patient’s diagnosis and treatment process (control flow) as well as event attributes value (data flow). It could predict the next activities in medical decision points, and implicitly integrates clinical guidelines into the prediction models. Taking into account medical knowledge, cultural differences, economic costs and therapeutic effects, our predictive models could achieve a higher accuracy, which could remind doctors avoid missing patients who may receive thrombolytic therapy. Moreover, although we take thrombolytic decision-making of stroke as an example, this method could be extended to other clinical decision support applications.

The first limitation of our approach is that we assumed the historical medical records can fully represent various situations inconsistent with the guidelines. Secondly, the relative contraindications and those patients with complex complications are not taken into account, even if they are diagnosed with acute ischemic stroke. Lastly, this method relies on the accuracy and completeness of the electronic medical records in the information system.

This current research is just based on one hospital, and the number of samples is relatively small. Therefore, we are planning to combine with the National Stroke Prevention and Control Project in China, to conduct training and validation on the data sets of multiple advanced stroke centers (large general hospitals), to prove that our method is universally applicable and that our models could be migrated. On the other side, since the cooperative hospital has only started the mechanical thrombectomy operation from the last 2 years, we also plan to establish a predictive model for mechanical thrombectomy in future.

## Data Availability

The dataset supporting the conclusions of this article is not available since the privacy of patients is included.

## Abbreviations

CGs: Clinical guidelines
CDSS: Clinical decision support system
RBR: Rule-based reasoning
CBR: Case-based reasoning
CVD: Cerebral Vascular Disease
EBM: Evidence-based medicine
GWTG: Get With The Guidelines
AIS: Acute ischemic stroke
rt-PA: Recombinant tissue plasminogen activator
MRA: Magnetic resonance angiography
DT: Decision tree
RF: Random forest
SVM: Support vector machines
GBRM: Generalized boosted regression models
EMR: Electronic Medical Record
ROC: Receiver Operating Characteristic
AUC: Area Under the ROC curve
FPR: False positive rate
TPR: True positive rate
